# Evolution of almond genetic diversity and farmer practices in Lebanon: impacts of the diffusion of a graft-propagated cultivar in a traditional system based on seed-propagation

**DOI:** 10.1186/s12870-018-1372-8

**Published:** 2018-08-06

**Authors:** Bariaa Hamadeh, Lamis Chalak, Geo Coppens d’Eeckenbrugge, Laure Benoit, Hélène I. Joly

**Affiliations:** 1 Lebanese Agricultural Research Institute, Fanar, Lebanon; 2 Université Montpellier 2, UMR CEFE, 34293 Montpellier Cedex, France; 3 Doctoral School of Sciences and Technologies, Lebanese University, Hadath, Lebanon; 4 CIRAD, UMR AGAP, Dynamiques de la Diversité, Sociétés et Environnements (DDSE), TA A-61/03 Avenue Agropolis, F-34398 Montpellier Cedex 5, France; 5 AGAP, Univ Montpellier, CIRAD, INRA, Montpellier SupAgro, Montpellier, France; 6 Faculty of Agriculture, Lebanese University, Dekwaneh, Lebanon; 7 CIRAD, UMR CBGP, F-34398 Montpellier, France; 8 CBGP, Univ Montpellier, CIRAD, INRA, IRD, Montpellier SupAgro, Montpellier, France

**Keywords:** Population genetics, *Prunus*, Agricultural practices, Almond, Vegetative propagation, Grafting, Genetic differentiation

## Abstract

**Background:**

Under cultivation, many outcrossing fruit tree species have switched from sexual reproduction to vegetative propagation. Traditional production systems have persisted, where cultivar propagation is based on a mixed reproductive system. For millenia, almond, *Prunus dulcis*, has been propagated by seeds. Almond grafting remained of little importance until recently. In Lebanon, both sexual and clonal reproductions are used for almond propagation. We used 15 microsatellite markers to investigate the effect of introducing graft-propagated cultivars and associated practices, on the structure of the genetic diversity among and within the two main Lebanese cultivars.

**Results:**

As expected, the sexually propagated cultivar Khachabi exhibited more genotypic and genetic diversity than the vegetatively propagated cultivar Halwani. It also exhibited lower differentiation among populations. The distribution of clones showed that propagation modes were not exclusive: farmers have introduced clonal propagation in the seed-propagated cultivar while they have maintained a diversity of genotypes within populations that were mostly graft-propagated. These practices are also important to avoid mate limitations that hamper fruit production in a self-incompatible species.

‘Khachabi’ is structured into two gene pools separated by the Lebanese mountains. As to ‘Halwani’, two different gene pools were introduced. The most ancient one shares the same geographic range as ‘Khachabi’; longtime coexistence and sexual reproduction have resulted in admixture with ‘Khachabi’. In contrast, the more recent introduction of the second gene pool in the Bekaa region followed an evolution towards more extensive clonal propagation of ‘Halwani’ limiting hybridizations. Furthermore, some pairs of geographically distant ‘Halwani’ orchards, exhibited low genetic distances, suggesting that a network of exchanges between farmers was effective on a large scale and/or that farmers brought clonal plant material from a common source.

**Conclusions:**

Almond diversification in Lebanon is clearly related to the evolution of propagation practices adapted to self-incompatible cultivars. The comparison between both cultivars demonstrated the genetic effects of the introduction of a new cultivar and the associated grafting propagation practices. Our study provided information to develop a strategy for in situ conservation of cultivars and to limit gene flow from introduced material to ancient orchards.

**Electronic supplementary material:**

The online version of this article (10.1186/s12870-018-1372-8) contains supplementary material, which is available to authorized users.

## Background

Population genetic structure of a crop plant species is strongly determined by their mating system and mode of dissemination. Allogamous seed reproduction maintains the crop evolutive potential because it produces genetically variable progenies able to cope with and adapt to changing environments [1–3]. Characteristically, natural populations of outcrossing species present high within-population diversity and low between-population differentiation for nuclear genes [4]. Long-lived perennials uphold significantly higher levels of genetic diversity within populations than annual plants [5, 6]. In a plant population, strict or partial clonal propagation results in a non-random distribution of genotypes, i.e., genetic sub-structuring [7]. High levels of clonal propagation lead to reduced genotypic diversity concomitant with an increase in population differentiation and heterozygosity [7, 8].

Under cultivation, many outcrossing fruit tree species have switched from sexual reproduction to vegetative propagation [9], and the cost of the resulting clonality is precisely an absence or reduction of sexual recombination [10]. Vegetative propagation, by way of grafts or cuttings, ensures that selected traits are reliably passed true-to-type onto the next generation [11]. This, together with large-scale cultivation of genetically uniform cultivars, has increased genetic vulnerability in intensive fruit production. However, less intensive production systems have persisted, where cultivar propagation is based on a mixed reproductive system, with two interlinked compartments corresponding respectively to sexually and clonally propagated plants. In the long run, farmer management of the aforementioned compartments will determine the evolution and the population genetic structure of the species. Investigating the dynamics of such mixed sexual / clonal systems on spatial and temporal scales helps clarify these long-term processes. However, although the mating system of most clonally propagated crops is documented, their sexual reproductive ecologies are poorly known [11].

The relationship between sexual recombination and genetic diversity works in both directions. While the suppression of sexual recombination reduces genotypic diversity, mate limitations conversely affect the potential for sexual recombination in monoclonal or polyclonal populations of obligate out-crossers. When the target of cultivation is a fruit, production can be ensured if the reproductive constraints are alleviated, for example by parthenocarpy, a suppression of the self-incompatibility system, or a shift to hermaphroditism in dioecious species [10, 11]. Nevertheless, in many fruit tree species, even economically important ones whose cultivars have been propagated for centuries (e.g. pear, apple, cherry), efficient outcrossing is necessary, and sexual or clonal cultivar reproduction practices do not only concern propagation but also production potential. In modern, graft-established, horticulture, fruit production is ensured by establishing biclonal or polyclonal orchards, or inter-planting a few trees from a compatible pollen-donor. Synchronous flowering, pollinator activity control and separate harvesting of the different cultivars are then further constraints. How this problem is managed in traditional horticultural systems has largely escaped the attention of crop evolutionists.

The eastern Mediterranean Basin is documented to be one of the major centers of origin for agriculture, with the first domestication events of cereals and fruit trees occurring in the Levant about 12,000 years B.P. [12]. Almond, *Prunus dulcis* (Miller) D.A. Webb (syn. *Amygdalus communis* L. and *P. amygdalus* Batsch.) [13], is considered to be one of the earliest domesticated nut-producing trees. It is a small deciduous self-incompatible tree, native to the Mediterranean region, where *P. dulcis* and other *Prunus* species still grow in the wild [13]. For millennia, almond trees have been propagated by seeds [14]; thus, cultivated populations were domesticated a long time prior to the development of grafting [15]. Almond tree grafting was already known by the first century AD. However, it was of little importance until the beginning of the nineteenth century, when grafting became the propagation method used to multiply selected genotypes [16]. Thus, while both sexual and clonal reproductions have been used for almond propagation in the Mediterranean region, the introduction of new improved and clonal cultivars in the form of grafts has become more and more frequent [16, 17]. However, little attention has been paid to the effect of these introductions and the associated grafting practices, on the structure of the genetic diversity both among and within cultivars.

Molecular tools such as nuclear microsatellite SSR (simple sequence repeat) analysis have been successfully used to assess genetic diversity as well as to reveal synonymous and erroneous labeling in cultivated *Prunus* fruit germplasm [18–22] and in other Mediterranean cultivated fruit species such as olive [23, 24] and fig [25]. To our knowledge, the differentiation among graft- or seed-propagated populations was only noted in a Tunisian apricot study [21]; its authors clearly distinguished two gene pools that are directly related to the different propagation modes of the Tunisian apricot cultivars. This study confirms the need to take into account the propagation mode when studying the structure and dynamics of genetic diversity. However, the genetic diversity and structure within ancient seed propagated and recent clonally propagated almond cultivars has not been compared so far. Furthermore, to our knowledge, previous studies have been carried out at the individual level and not at the population level, except for Delplancke et al. [26] and Wang et al. [27]. In these studies, genotyping was used to characterize clonally and sexually propagated cultivars, identified by the genotype of a single individual. In our study we set out to evaluate the evolutionary process at the population level and carried out our sampling strategy accordingly.

Spontaneous populations of *P. dulcis* and its close wild relatives, *P. orientalis* (Mill.) Koehne and *P. spartioides* (Spach) C.K. Schneid., are still found in Lebanon [28–30]. Cultivated *P. dulcis* is present in different agro-climatic areas and is part of the Lebanese landscape. Old almond trees are still found over a wide range of elevations (0 to 1600 m). So far, studies on the diversity of almond in Lebanon have only concerned morphological traits [31] and the identification of almond cultivars [18]. These studies give a preliminary view of the wide distribution of some cultivars across the country and indicate greater varietal diversity in home gardens than in modern orchards. Using nuclear and chloroplastic SSR markers, gene flow were identified in both directions between cultivated almond species and *P. orientalis,* present in Lebanon, Syria and Turkey [26]. Moreover, the Levant was identified as a center of diversification for cultivated *P. dulcis*, since it is an important reservoir of almond genetic diversity [32].

Almond has not only been cultivated under widely divergent ecological conditions, but also the history of almond cultivation in the country is long-standing. Two main horticultural forms are recognized by farmers, based on fruit shell hardness. The most common and oldest cultivar is known as ‘Khachabi’, referring to its fruit shell ‘as hard as wood’; for centuries, it has been cultivated and reproduced by seeds, maintaining a high level of morphological variability. Since the Ottoman period (16th to early twentieth century), many cultivars have been introduced to the country from different origins. Among them, the cultivar now called ‘Halwani’, was probably introduced to Lebanon during the last century; it has been widely accepted and mostly propagated by grafts, mainly in mono-varietal populations [33].

Questions arise regarding the genetic diversity of ‘Khachabi’ (common hard shell cultivar) and ‘Halwani’ (introduced soft shell cultivar), as well as their relationships and internal structure, taking into account the influence of their respective propagation modes. Nowadays, sexual reproduction is still important in almond populations and this importance depends largely on whether farmers incorporate sexual progeny into their stocks of grafting material. This practice is common in many traditional farming systems, and has probably been continued from the origin of domestication up to our time. For the present study, we sampled a large range of cultivated individuals from different agro-climatic areas in Lebanon, and analyzed them with a set of 15 SSRs selected for their reproducibility and polymorphism in almond and other *Prunus* species, with the aim of: (1) characterizing the pattern of genotypic and genetic structure of each cultivar, ‘Khachabi’ and ‘Halwani’, and (2) assessing the influence of the propagation mode on these patterns.

## Methods

### Survey and sampling

Field surveys were undertaken in 2009–2010 throughout Lebanon with the aim of collecting samples representative of the most common almond cultivars, ‘Khachabi’ and ‘Halwani’. We followed farmer’s determinations in the assignation of sampled trees to a given cultivar; nomenclature was usually based on shell hardness. Farmers usually propagated hard shell almonds from seed and soft ones by grafting. According to farmers, out of 331 ‘Khachabi’ individuals, 301 were propagated by seeds, and the 30 individuals from the youngest population were propagated by grafting. Out of the 285 ‘Halwani’ individuals, 255 were propagated by grafting and the 30 individuals from the oldest population were propagated either by grafting or seeds. In all, 14 ‘Khachabi’ and 11 ‘Halwani’ populations were sampled, covering the four major agro-climatic zones (Table 1; see Additional file 1: Figure S1 presents the locations of the collected populations). Each sampled ‘Khachabi’ orchard only contained ‘Khachabi’ trees but the presence of rare individuals from other almond cultivars could sometimes be observed in ‘Halwani’ orchards. From the information gathered from farmers, we realized that, in localities of traditional almond production, orchards were either still constituted of the original cultivar Khachabi, or they had been rejuvenated by grafting most trees with ‘Halwani’ scions. In other localities, ‘Halwani’ had been established directly in new orchards. Our population approach led us to select orchards on the availability of a large enough number of individuals of a given cultivar.

**Table 1:** Populations collected throughout Lebanon

Population name	Cultivar	Eco-geographical zone	Code	n	NDD	EDD	Age of plantation (years)
Bire	Halwani	Bekaa	HB_Bi	23	34.5872	36.2391	25
Ferzol	Halwani	Bekaa	HB_Fe	33	33.8884	35.9396	25
Nabi Ayla	Halwani	Bekaa	HB_NA	30	33.8819	35.9569	25
Zighrine	Halwani	Bekaa	HB_Ze	16	34.4261	36.3528	25
Amchit	Halwani	Mount Lebanon	HM_Am	35	34.1475	35.6464	48
Maasriti	Halwani	Mount Lebanon	HM_Ma	30	33.7483	35.6347	30
Jeita	Halwani	Mount Lebanon	HM_Je	30	33.9533	35.6478	70
Bchannine	Halwani	North Lebanon	HN_Bc	35	34.3483	35.8859	20
Btaaboura	Halwani	North Lebanon	HN_Bt	25	34.2742	35.7622	20
Deir Qanoun el Nahr	Halwani	South Lebanon	HS_DQ	25	33.3000	35.3136	20
Tanbourit	Halwani	South Lebanon	HS_Ta	15	33.5161	35.4153	20
Bakkifa	Khachabi	Bekaa	KB_Ba	27	33.4933	35.8192	60
Bire	Khachabi	Bekaa	KB_Bi	20	33.5842	35.8197	40
El Mhaidtheh	Khachabi	Bekaa	KB_EM	30	33.5569	35.8117	60
Irsal 2	Khachabi	Bekaa	KB_Ir	15	34.1886	36.3923	35
Kamed el Lawz	Khachabi	Bekaa	KB_KL	33	33.6203	35.8214	60
Lucy	Khachabi	Bekaa	KB_Lu	35	33.6453	35.8383	60
Shaat	Khachabi	Bekaa	KB_Sh	18	34.1422	36.2311	30
Assia	Khachabi	North Lebanon	KN_As	25	34.2189	35.7856	50
El Qalamoun	Khachabi	North Lebanon	KN_EQ	16	34.3872	35.7864	70
Baraachit	Khachabi	South Lebanon	KS_Ba	30	33.1761	35.4433	50
Blida	Khachabi	South Lebanon	KS_Bl	30	33.1400	35.5147	50
Chebaa	Khachabi	South Lebanon	KS_Ch	23	33.3475	35.7492	80
Mays al Jabal	Khachabi	South Lebanon	KS_MJ	18	33.1686	35.5242	37
Tanbourit	Khachabi	South Lebanon	KS_Ta	30	33.5161	35.4153	30

n, Number of collected individuals

### Microsatellite amplification

Total genomic DNA was extracted from silica-dried leaves using Extract-N-Amp™ Plant PCR Kits (SIGMA - Aldrich, St. Louis, MO, USA) abiding by the supplier’s instructions. Fifteen nuclear SSR markers previously selected for studying the genetic diversity of *Prunus* species in the Mediterranean region were used for this study: UDP96–001, UDP96–018, UDP96–003, UDP97–401, UDP98–408, UDP98–409, pchgms1, pchgms3, BPPCT017, BPPCT001, BPPCT007, BPPCT025, BPPCT036, CPSCT018, CPDCT045 [34–38]. We amplified these 15 SSR markers into three multiplexed PCRs, using one of the FAM, HEX or NED fluorophore-labeled primers (PE Applied Biosystems, Warrington, UK). Multiplexed PCRs were carried out with the Extract-N-Amp PCR Ready Mix (SIGMA - Aldrich, St. Louis, MO, USA) in a final volume of 10 μl, containing 5 μl of a SIGMA Master Mix 2X, 0.4 μl of primer mix at 5 μM, 1.6 μl of ultrapure water and 1 μl of template dNTPs. 3 μl of PCR product was mixed with 15 μl of formamide and 0.2 μl of Genescan™ 500 LIZ size standard (Applied Biosystems, Foster City, USA), and GeneScan was performed with the ABI PRISM 3130 XL 16 capillary-sequencer (Applied Biosystems, Foster City, USA).

### Genotyping and data organization

Allele size was read independently by two investigators using GEMEMAPPER 4.0 (Applied Biosystem, Foster City, USA). Genotyping errors were evaluated by checking the reproducibility of migrations using samples replicated on the different plates; in all, 95.1% of tested individuals gave the same allele size (i.e., the error rate was under 0.05).

Redundant genotypes were searched both within cultivars and among populations of both ‘Khachabi’ and ‘Halwani’ to identify multi-locus genotypes (MLGs) using GENCLONE 2.0 software [39]. Individuals with identical MLGs were considered as a clone. Different datasets were used for different analyses at different levels: global, cultivar and populations. Datasets were constructed using two different matrices: a matrix with a total of 615 individuals that will be referred to as the N dataset and a matrix with a single copy of redundant MLGs conserved within each population (with a total of 509 individuals) that will be referred to as the G dataset.

### Data analysis

#### Genotypic diversity

The discriminant power of the 15 markers used to differentiate MLGs present in the sample was explored by plotting the number of loci versus the maximum number of MLGs detected for all datasets and for each cultivar; values were calculated using a re-sampling procedure (3000 permutations) implemented in GENCLONE. The relevance of marker information was described by the number of alleles (Na) implemented in the software GENALEX 6.5.1 [40] and the polymorphic information contents [41] $\left(\mathrm{PIC}= 1-{\sum}_{\mathrm{i}}{p}_i^2-{\sum}_{i,j}{p}_i^2\right.{p}_j^2$, where *p*_*i*_ and *p*_*j*_ are the frequency of the *i*th and *j*th alleles; implemented in CERVUS 3.0.7 software [42].

Genotypic richness was calculated following the corrected formula [43], R = (G-1) / (N-1), where G is the number of distinct genotypes and N is the number of individuals sampled in a population, implemented in GENCLONE. The probability of observing two identical MLGs in each population (PI) was calculated using the GENALEX software.

Genotypic linkage disequilibrium (LD) between loci was calculated on the G dataset to avoid its overestimation; the coefficients of correlation (r) between loci were calculated within the G dataset, each cultivar and the distinct population subsets. Testing for genotypic linkage disequilibrium (LD) using the log likelihood ratio statistic test (G-test) was performed with the software GENEPOP 4.2 [44, 45], available on the web (http://genepop.curtin.edu.au/genepop_op2.html). Exact *P-*values were estimated using the Markov Chain algorithm with 10,000 dememorization steps, 1000 batches and 10,000 iterations.

We conserved a single copy of each MLG (G dataset) to identify the numbers of different alleles between each pair of compared MLGs in each cultivar. The histograms of pairwise comparisons were constructed using simple matching of pairs of MLGs using Unweighted Arithmetic Average, implemented in the CLUSTERING CALCULATOR program developed by J. Brzustowski (http://www.biology.ualberta.ca/jbrzusto/cluster.php).

#### Null alleles, hardy-Weinberg equilibrium

The existence and frequency of null alleles were tested (on the N dataset) using the Expectation Maximization (EM) algorithm [46] implemented in GENEPOP (http://genepop.curtin.edu.au/genepop_op8.html). Deviations from Hardy-Weinberg equilibrium (HWE) were tested using both alternative hypotheses of a deficit or of an excess of heterozygotes for each locus and across loci, at population and at cultivar levels using Fisher method implemented in GENEPOP (http://genepop.curtin.edu.au/genepop_op1.html). The Hardy-Weinberg Equilibrium tests were calculated based on both N and G datasets.

#### Genetic diversity

Rarefied allelic richness and private allelic richness were computed on the N dataset using the rarefaction method implemented in ADZE 1.0 [47], with a minimum sample size of G = 15 genotypes. The small size of some samples when eliminating redundant MLGs from the populations limited the estimation of genetic diversity parameters. Genetic diversity was estimated, for each locus, at the population and cultivar levels. Values were estimated using the unbiased expected heterozygosity UHe = 2 N / (2 N-1)*He (where He = *1-Σpi*^*2*^, and *pi* is the frequency of the *i*th allele in the population) implemented in GENALEX, the observed heterozygosity Ho and the Wright’s fixation index (Fis = *1-Ho/He*) [48], implemented in GENETIX 4.05.2 [49]. Differences in mean diversities for each parameter were compared between populations and between both cultivars using the Mann-Whitney U-test implemented in XLSTAT-Pro 7.5 (Addinsoft 1995–2015). The Fis over loci and populations were calculated using the N dataset to better describe the sampled data; we also calculated the Fis for populations with sample size (≥15 individuals) after elimination of the redundant genotypes (G datasets). Confidence intervals at 95% were calculated using 10,000 bootstraps (GENETIX software). Wilcoxon’s signed rank test, implemented in XLSTAT-Pro 7.5, was performed for comparisons of heterozygosity expected under HWE and to test differences in Fis values based on N and G matrices between cultivars.

#### Population differentiation and organization of genetic diversity

The genetic differentiation among collected populations was calculated for the N dataset. Null alleles may lead to overestimate population genetic differentiations as measured by Fst [50]. To calculate pairwise genetic distances between each pair of populations we therefore used corrected Fst values using the ENA (excluding null alleles) method, and corrected values of the Cavalli-Sforza and Edwards [51] genetic distance (Dc) using the INA (including null alleles) method, implemented in FreeNA [50]. Geographic distances between populations were estimated based on the populations coordinates using the following formula: Ln(1 + Geographic Distance) implemented in GENALEX. In order to test isolation by distance (IBD) within each cultivar, Mantel tests [52] were conducted to measure Pearson’s correlation using the GENALEX software; significance at α = 0.05 was determined using 1000 randomizations of the elements of the genetic distance (Dc) x geographic distance matrix.

Two different approaches were used to infer the population structure of the whole dataset. The first was a Bayesian clustering approach assuming Hardy-Weinberg and linkage equilibrium, as implemented in the STRUCTURE 2.3.4 software [53]. This analysis benefited from the Montpellier Bioinformatics Biodiversity platform services (http://mbb.univ-montp2.fr/MBB/subsection/onlineTools.php?section=2). STRUCTURE was run on the whole data, using both the G and the N datasets, to infer population structure in K genetic clusters and assign individuals to those clusters. Structure was run also on partitioned subsets, for each cultivar, in order to investigate lower levels of structure. Individuals were assigned by probability to a cluster, or jointly to two or more clusters if their genotypes indicated that they were admixed. Individuals for which more than 85% of alleles were assigned to a cluster were considered to belong to a single cluster. To infer posterior probabilities of K, we ran a series of independent runs with different user-defined values of K ranging from 1 to 10. The analysis was run ten times for 10^7^ iterations of MCMC after a burn-in period of 10^6^ based on pre-defined population genetics admixture and no linkage models. To identify the number of K clusters explaining the observed genetic structure we used the statistic parameters defined by Evanno et al. [54] based on the rate of change in the log probability of data between successive K values, implemented in a web-based program, STRUCTURE HARVESTER 0.6.94 [55]. The CLUMPP [56] output served to DISTRUCT 1.1 software [57] to graphically represent the estimated population structure, according to the geographic proximity and ecological region of each cultivar. Each individual was represented by a thick line, which was partitioned into K colored segments, representing the individual’s estimated membership fractions in the K clusters.

The population genetics model in STRUCTURE, relies on restrictive explicit assumptions (populations at Hardy-Weinberg equilibrium and linkage equilibrium) which are likely to be violated for cultivated species, especially those exposed to clonal propagation. The presence of substantial null allele frequencies may also affect the estimation of the population differentiation [50]. Hence, we choose the discriminant analysis (DA) of principal components (DAPC; [58]) as a second approach, as it is a non-model clustering method. DAPC was performed to identify and describe clusters of genetically related individuals from G dataset, using the Adegenet 2.0.1 package [59] implemented in R software [60]. This multivariate analysis seeks linear combinations of the original variables (alleles), which maximize between-group variation and minimize within-group variation [58].

DAPC is a multivariate method that relies on data transformation using a principal component analysis as a prior step to the DA. This prior transformation ensures that variables subjected to the DA are uncorrelated. The identification of genetic groups was achieved using K-means [61], a clustering algorithm which finds a given number (K) of groups maximizing the differentiation between groups. To identify the optimal number of groups, K-means was run sequentially with increasing values of K, in a loop of 20 repetitions, and the optimal number of K groups was identified by its lowest Bayesian Information Criterion (BIC) [58]. We executed the cross-validation to provide an objective optimization procedure for identifying the number of PC axes to retain, since the number of PC axes can have a substantial impact on the results of the global DAPC analysis [58]. The cross-validation was carried out using the function xvalDapc with 100 repetitions. The cross-validation method was also used to provide individuals’ assignment to groups as well as a visual assessment of between-population differentiation and admixture. Based on the optimal number of clusters and the retained discriminant functions, the DAPC analysis was run and derived probabilities for each individual membership in each of the different groups. Admixed individuals were identified at a threshold of 85% of assignation to a group.

## Results

### Information related to SSR markers

The 15 SSR loci revealed a total number of 375 alleles, ranging from six alleles at the BPPCT036 locus to 41 at the CPDCT045 locus (see Additional file 2: Table S1). All SSR markers, except BPPCT007, were in Hardy-Weinberg disequilibrium (Fis ≠ 0) for N and G datasets. At the whole dataset level, four out of 15 loci were supposed to exhibit null alleles; the estimated frequency of these alleles ranged from 15% for UDP98–409 up to 27% for CPDCT045. Seven loci showed null alleles with frequencies over 10% for ‘Khachabi’; among them, two loci (UDP97–401 and CPDCT045) exhibited also null alleles for ‘Halwani’ (see Additional file 2: Table S1).

In all, 105 pairwise comparisons between loci were conducted over all datasets without redundant MLGs (see Additional file 3: Table S2). The coefficient of correlation was *r* = 0.75 for the whole dataset. At the cultivar level, significant genotypic LD (*P* < 0.05) was identified for ‘Khachabi’ *r* = 0.22 (and 95% of pairwise comparisons were in LD) while for ‘Halwani’ *r* = 0.43 (and 74% of pairwise comparisons were in LD) (Tables 2; see Additional file 3: Table S2).

**Table 2:** Genetic and genotypic diversity measurements

Pop	Genotypic Diversity				% LD(G)	Genetic diversity					
	N	G	R	PI (N)		Ar+	Pa+	Ho (N)	He (N)	Fis (N)	Fis (G)#
Halwani	284	195	0.69	1.20E-13	95	4.80 (0.36)	2.01 (0.31)	0.67 (0.07)	0.66 (0.04)	−0.03	0
HB_Bi	23	8	0.32	4.40E-11	42	3.60 (0.25)	0.02 (0.01)	0.70 (0.08)	0.62 (0.03)	−0.12*	–
HB_Fe	33	14	0.41	7.60E-08	77	2.89 (0.20)	0.10 (0.07)	0.62 (0.12)	0.47 (0.06)	−0.34*	–
HB_NA	30	19	0.62	2.20E-12	83	3.93 (0.25)	0.04 (0.02)	0.74 (0.08)	0.64 (0.05)	−0.15	−0.08*
HB_Ze	16	7	0.4	1.50E-06	14	2.12 (0.18)	0.00 (0.00)	0.54 (0.12)	0.42 (0.06)	−0.28*	–
HM_Am	29	27	0.93	2.60E-08	20	2.86 (0.29)	0.12 (0.08)	0.63 (0.09)	0.49 (0.06)	−0.33*	−0.30*
HM_Ma	30	21	0.69	6.10E-09	21	2.96 (0.24)	0.05 (0.03)	0.75 (0.09)	0.52 (0.05)	−0.45*	−0.39*
HM_Je	30	29	0.97	1.60E-17	52	6.10 (0.40)	0.21 (0.06)	0.66 (0.04)	0.76 (0.03)	0.13*	0.14*
HN_Bc	32	22	0.68	6.80E-11	70	3.65 (0.32)	0.03 (0.01)	0.75 (0.08)	0.59 (0.05)	−0.28	−0.21*
HN_Bt	23	23	1	7.70E-12	43	3.91 (0.51)	0.20 (0.09)	0.62 (0.08)	0.60 (0.07)	−0.03	−0.03
HS_DQ	23	14	0.59	6.30E-08	50	2.93 (0.25)	0.02 (0.02)	0.73 (0.11)	0.48 (0.06)	−0.56*	–
HS_T a	15	10	0.64	7.90E-09	18	3.10 (0.24)	0.07 (0.03)	0.52 (0.08)	0.51 (0.06)	−0.06*	–
Khachabi	331	314	0.95	1.80E-25	74	8.28 (0.50)	5.48 (0.45)	0.68 (0.05)	0.86 (0.03)	0.20	0.20*
KB_Ba	26	26	1	2.30E-21	3	7.23 (0.47)	0.23 (0.07)	0.64 (0.05)	0.84 (0.03)	0.24	0.24*
KB_Bi	20	20	1	4.40E-16	21	6.08 (0.41)	0.29 (0.08)	0.70 (0.06)	0.74 (0.03)	0.05	0.05*
KB_EM	30	30	1	2.80E-21	13	7.47 (0.49)	0.22 (0.06)	0.73 (0.05)	0.82 (0.03)	0.12	0.12*
KB_Ir	15	15	1	3.70E-18	33	6.31 (0.47)	0.41 (0.14)	0.72 (0.06)	0.79 (0.04)	0.10*	0.10*
KB_KL	30	29	0.97	1.50E-20	13	7.01 (0.46)	0.26 (0.07)	0.70 (0.05)	0.83 (0.02)	0.15	0.15*
KB_Lu	30	30	1	1.10E-20	14	6.92 (0.43)	0.31 (0.06)	0.73 (0.05)	0.83 (0.03)	0.13	0.13*
KB_Sh	18	18	1	1.40E-17	37	6.19 (0.37)	0.23 (0.11)	0.63 (0.06)	0.78 (0.04)	0.18	0.18*
KN_As	16	16	1	3.10E-19	20	6.92 (0.53)	0.41 (0.12)	0.62 (0.07)	0.80 (0.04)	0.22	0.22*
KN_EQ	16	16	1	8.20E-19	14	6.83 (0.52)	0.56 (0.13)	0.62 (0.06)	0.79 (0.05)	0.22*	0.22*
KS_Ba	30	30	1	7.20E-21	22	7.20 (0.48)	0.32 (0.08)	0.71 (0.06)	0.82 (0.03)	0.12	0.12*
KS_Bl	29	29	1	2.40E-19	18	6.71 (0.42)	0.18 (0.06)	0.67 (0.05)	0.80 (0.03)	0.16	0.16*
KS_Ch	23	21	0.91	1.30E-20	25	7.14 (0.47)	0.37 (0.12)	0.71 (0.05)	0.83 (0.03)	0.14	0.15*
KS_MJ	18	17	0.94	2.90E-17	19	5.91 (0.40)	0.12 (0.05)	0.71 (0.06)	0.77 (0.04)	0.08	0.07*
KS_T a	30	18	0.59	1.80E-15	52	5.32 (0.30)	0.13 (0.04)	0.65 (0.08)	0.73 (0.03)	0.11*	0.15*

N, Number of samples; G, Number of distinct genotypes; R, Proportion of distinct genotypes = (G-1)/(N-1); PI, probability of identity of two genotypes; %LD: percentage of genotypic linkage disequilibrium based on a single MLG copy; Ar^+^, average number of alleles per locus; Pa^+^, mean number of private alleles per locus; Ho, observed heterozygosity; He, unbiased expected heterozygosity; Fis, inbreeding coefficient

^+^Standardized population at G = 15 using rarefaction method

^#^Fis calculated for sample size over 15 individuals

*Significant departure from Hardy-Weinberg equilibrium (*P* ≥ 0.05)

X_(N)_ Values calculated based on N matrix

X_(G)_ Values calculated based on G matrix

Standard errors are in brackets

The 15 microsatellite loci allowed to distinguish 314 MLGs out of 331 individuals distributed over the 14 ‘Khachabi’ populations, whereas 195 distinct MLGs in a total of 284 sampled individuals were distributed over the 11 ‘Halwani’ populations (Table 2). Our results showed that the set of 15 SSR loci allowed an efficient estimation of the true number of MLGs; the total number of MLGs was reached for 14 loci for the whole dataset and already for 11 and 12 loci for ‘Khachabi’ and ‘Halwani’ respectively (see Additional file 4: Figure S2).

### Genotypic diversity

The mean genotypic diversity value for cultivar Khachabi was significantly higher than the value reported for cultivar Halwani (R _Khachabi_ = 0. 95 and R _Halwani_ = 0.69; U-test, *P* = 0.000; α = 0.05). Populations from ‘Khachabi’ exhibited higher genotypic diversity levels than populations from ‘Halwani’, except for the ‘Khachabi’ KS_Ta population, due to the presence of three farmer-selected clones (*R* = 0.59) (Table 2).This indicates the presence of more redundant MLGs in ‘Halwani’ populations, as compared to ‘Khachabi’ populations. However, among ‘Halwani’ populations, HM_Am and HM_Je exhibited only two and one redundant MLGs respectively (corresponding *R* = 0.93 and 0.97, respectively) and no redundant MLGs were identified in the HN_Bt population (*R* = 1.00).

**Fig. 1:**
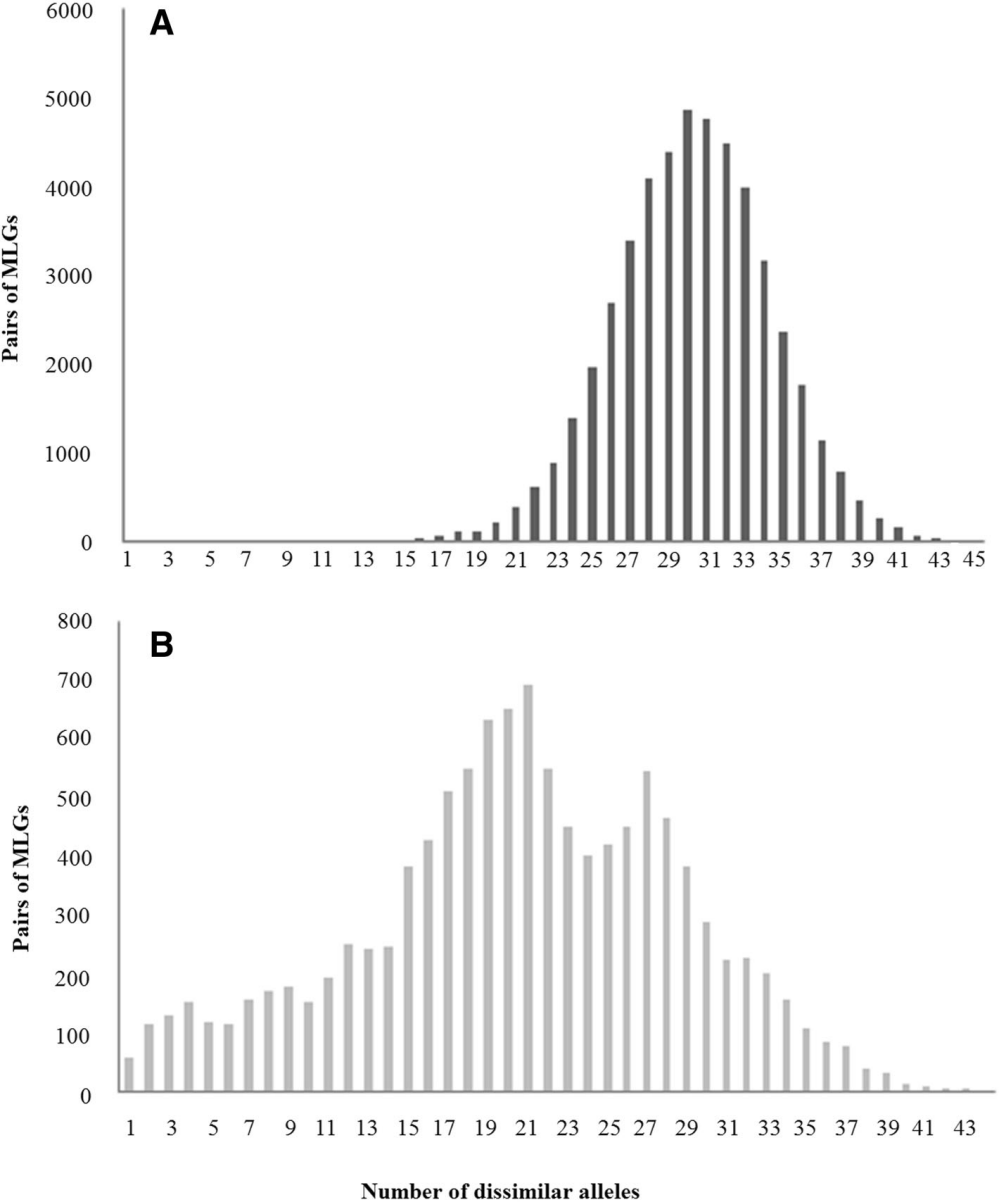
Distribution of the pairwise number of allele differences among MLGs. **a** Unimodal curve for ‘Khachabi’ genotypes; 48,826 pairwise comparisons, scale range from 0 to 6000 **b**) A bimodal shaped curve for ‘Halwani’; 11,264 pairwise comparisons after elimination of the 33 individuals assigned at 85% to ‘Khachabi’, Scale range from 0 to 800

We computed the distribution of the number of allelic differences between individuals (N dataset) for each cultivar; individuals were plotted after suppression of the redundant MLGs. In ‘Khachabi’, 48,798 pairwise comparisons ranged from 11 to 45 allelic differences with a peak at 30 differences, while only 28 pairwise comparisons were differing by 1 to 10 alleles (Fig. 1a). Individuals from cultivar Halwani were plotted after elimination of individuals assigned by STRUCTURE (see below) to the cluster that mainly comprised ‘Khachabi’ individuals (Fig. 1b). 11,264 pairwise comparisons for the ‘Halwani’ MLGs (Fig. 1b) displayed a continuity of values ranging from 1 to 43 differences with a multimodal curve; two major peaks, at 21 and 27, were observed. In comparison to ‘Khachabi’ a total of 1542 pairwise comparisons differed by up to 10 alleles.

Forty-two redundant MLGs were defined as clones; three were observed in the clonally propagated ‘Khachabi’ population KS_Ta (data not shown) while 36 were observed in 10 of the 11 ‘Halwani’ populations. Among these, 24 were private (associated to one population) and 12 clones were found across different populations. Some clones were shared among geographically distant populations of ‘Halwani’.

### Genetic diversity

To better describe the genetic diversity existing within each cultivar and population, we decided to conduct analyses using the N dataset. ‘Khachabi’ exhibited a larger number of alleles than ‘Halwani’ with 361 and 221 alleles, respectively (Table 2).

Genetic diversity parameters were calculated over loci and the values are presented with their standard errors (Table 2). The mean rarefied allelic richness (Ar) and the private allelic richness (Pa) were significantly higher (U-test, *P* < 0.0001; α = 0.05) for ‘Khachabi’ (Ar = 8.28 ± 0.50 and Pa = 5.48 ± 0.45), as compared to ‘Halwani’ (Ar = 4.80 ± 0.36 and Pa = 2.01 ± 0.31). The mean genetic diversity was significantly higher for ‘Khachabi’ (He = 0.86 ± 0.03) than for ‘Halwani’ (*H*E = 0.66 ± 0.04) (*U*-test, *P* = 0.002; α = 0.05). Cultivar Khachabi also displayed a significant deficit in heterozygosity (Fis = 0.20) while cultivar Halwani had no deficit in heterozygosity (Fis = − 0.03). The comparison of the fixation index (Fis) (N dataset) exhibited high differences between ‘Khachabi’ and ‘Halwani’ (Wilcoxon’s test, *P* < 0.0001; α = 0.05). The confidence intervals of Fis values are presented in (Additional file 5: Table S3).

Different genetic diversity levels were also observed among populations, the lowest number of alleles for the ‘Halwani’ populations being in HB_Ze (Ar = 2.12 ± 0.18) and the highest number in the HM_Je population (Ar = 6.10 ± 0.40) (Table 2). The number of private alleles (Pa) ranged from 0 to 0.21. Ten out of eleven ‘Halwani’ populations exhibited an excess of heterozygotes with a significant departure from Hardy-Weinberg equilibrium (*P* ≥ 0.05). The number of rarefied private alleles (Pa) ranged from 0.12 to 0.56. All ‘Khachabi’ populations exhibited a high deficit in heterozygosity (*P* ≤ 0.05) (Table 2).

### Population genetic structure

**Fig. 2:**
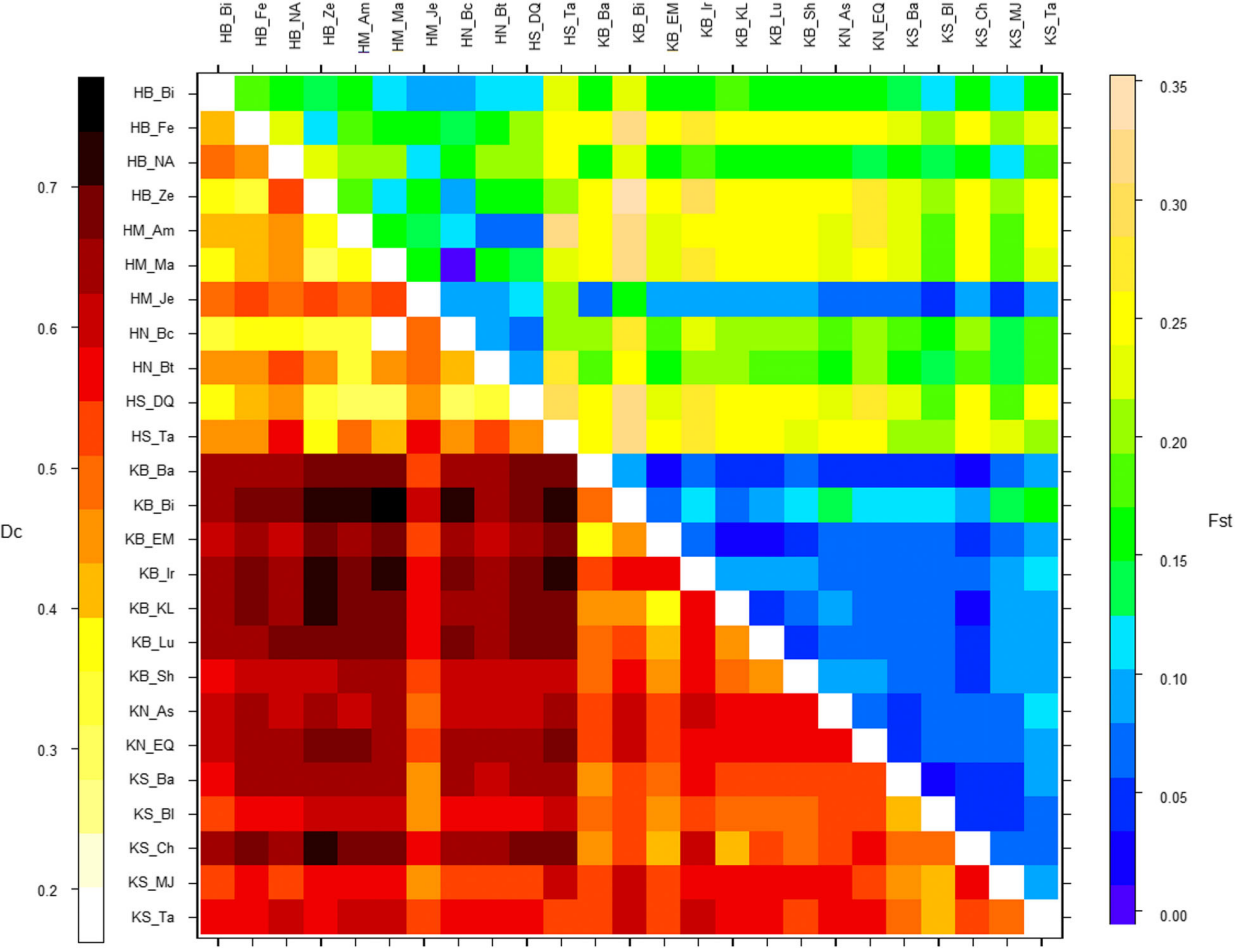
Heat map for pairwise genetic distance between populations. Upper part: Fst; lower part: Dc (Cavalli-Sforza). Values were calculated with 1000 permutations. Values are significant at *P* > 0.001. Genetic differentiation is: Low to moderate 0.02 < x < 0.15 (in blue), high genetic differentiation 0.15 < x < 0.25 (in green) and very high > 0.25 (in yellow). Genetic distance (Dc) is: Low to moderate 0.02 < x < 0.4 (white to yellow), high genetic distance 0.4 < x < 0.8 (red to black)

Genetic differentiation (Fst) varied from 0.03 to 0.12 among ‘Khachabi’ populations, and from 0.01 to 0.31 among ‘Halwani’ populations; the highest Fst observed among populations from both cultivars was 0.33 (between HB_Ze and KB_Bi) (Fig.2). The pairwise estimate values of Dc varied from 0.37 to 0.62 among ‘Khachabi’ populations, and from 0.20 to 0.56 among ‘Halwani’ populations, and from 0.44 to 0.74 among populations from both cultivars (Fig.2). The Mantel test revealed a pattern of isolation-by-distance in ‘Khachabi’ (R2 = 0.20, *P* = 0.006) but not in ‘Halwani’ (R2 = 0.04, *P* = 0.124) (see Additional file 6: Figure S3).

**Fig. 3:**
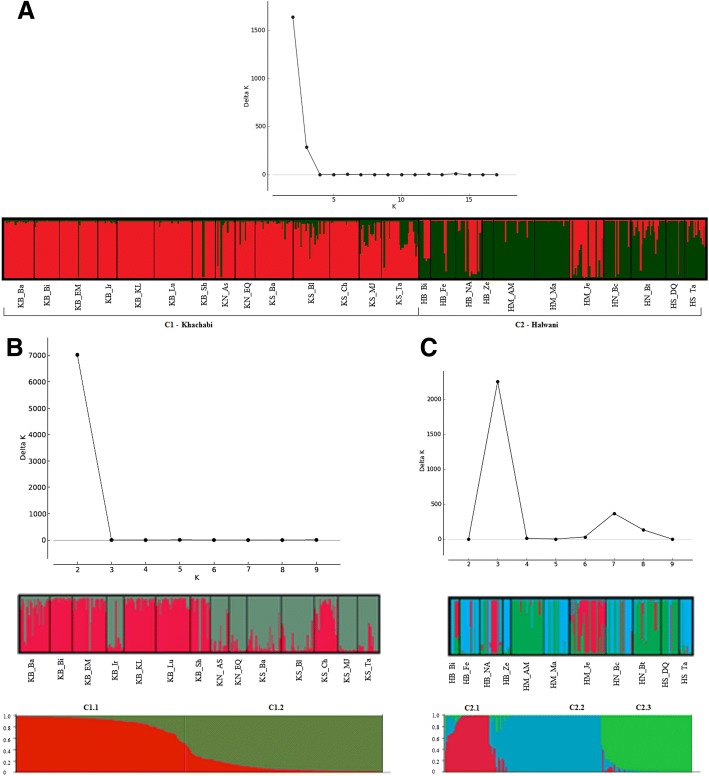
Genetic Structure of almond genotypes estimated by STRUCTURE. **a** DISTRUCT representation of 14 ‘Khachabi’ and 11 ‘Halwani’ populations (509 genotypes). K = 2, ΔK = 1640.26 and H′ = 0.998; **b**) DISTRUCT representation of 14 ‘Khachabi’ populations (314 genotypes); ΔK graph: K = 2, ΔK = 7024.5 and H’ = 0.999; **c**) DISTRUCT representation of 11‘Halwani’ populations (195 genotypes); K = 3, ΔK = 2251.1 and H’ = 0.999

The STRUCTURE analysis on the overall G dataset revealed the highest ΔK for K = 2 (ΔK = 1640.26, H′ = 0.999); 92% of ‘Khachabi’ individuals were assigned to cluster 1 (in red) and 81% of ‘Halwani’ individuals were assigned to cluster 2 (in green) (Fig. 3a). Admixed individuals among both clusters were observed in both cultivars at a threshold of assignation of 85%. The lowest percentages of assignment to the cultivar Halwani were observed in HB_Bi, HB_NA and HM_Je populations with 57, 58 and 34% of individuals assigned to cluster 2, respectively. On the whole sample, the STRUCTURE analysis assigned 117 ‘Halwani’ individuals, belonging to 29 clones, to cluster 2. Fourteen individuals from HB_NA belonging to five clones and two individuals from the HB_Fe ‘Halwani’ populations, belonging to one clone, were assigned to cluster 1. Eight individuals from HB_Bi belonging to two clones and three individuals from the HB_NA belonging to one clone were admixed among both clusters.

**Fig. 4:**
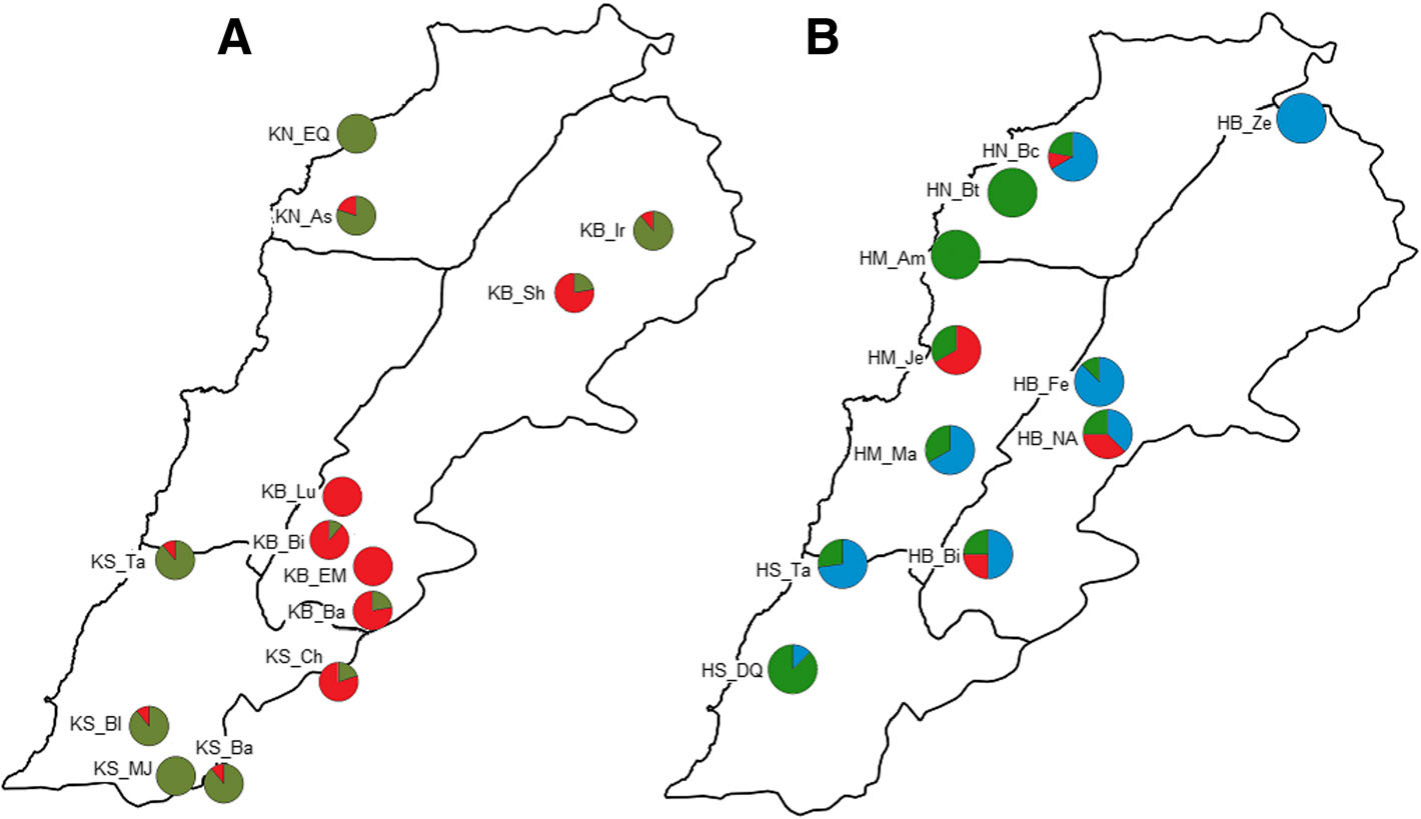
Geographical distribution of STRUCTURE assignment, each population is represented in a pie chart. **a** K = 2 for ‘Khachabi’ populations; **b**) K = 3 for ‘Halwani’ populations. Full names of the abbreviations for the populations are shown in Table 1

The STRUCTURE analysis of ‘Khachabi’ assigned its 314 MLGs into two clusters (K = 2, ΔK = 7024.5 and H’ = 0.999) (Fig. 3b). Populations belonging to these clusters were geographically differentiated (Fig. 4a). The first cluster (C1.1, red) comprised the populations of the Bekaa region, except for the population KB_Ir; the nearby population from the South, KS_Ch, belonged also to this cluster. The second cluster (C1.2, olive green) was composed of populations from the South and North regions and the KB_Ir population from the Bekaa region. Some individuals were admixed among both clusters with percentages of assignation under 85%.

The STRUCTURE analysis of the populations named as ‘Halwani’ assigned the 195 MLGs of this cultivar into three clusters (K = 3, ΔK = 2251.1 and H’ = 0.999) (Fig. 3c). As expected, the first cluster (C2.1, red) included the individuals which were re-assigned to ‘Khachabi’ in the global analysis (C1, Fig. 3a). The other individuals were assigned into two clusters (C2.2 in blue and C2.3 in green, Fig. 3c). The geographic differentiation of populations was not as clear-cut as for ‘Khachabi’ populations (Fig. 4b). Individuals from a same population were mainly assigned to the same cluster. Individuals from HN_Bt and HM_AM exhibited admixture between both clusters.

**Fig. 5:**
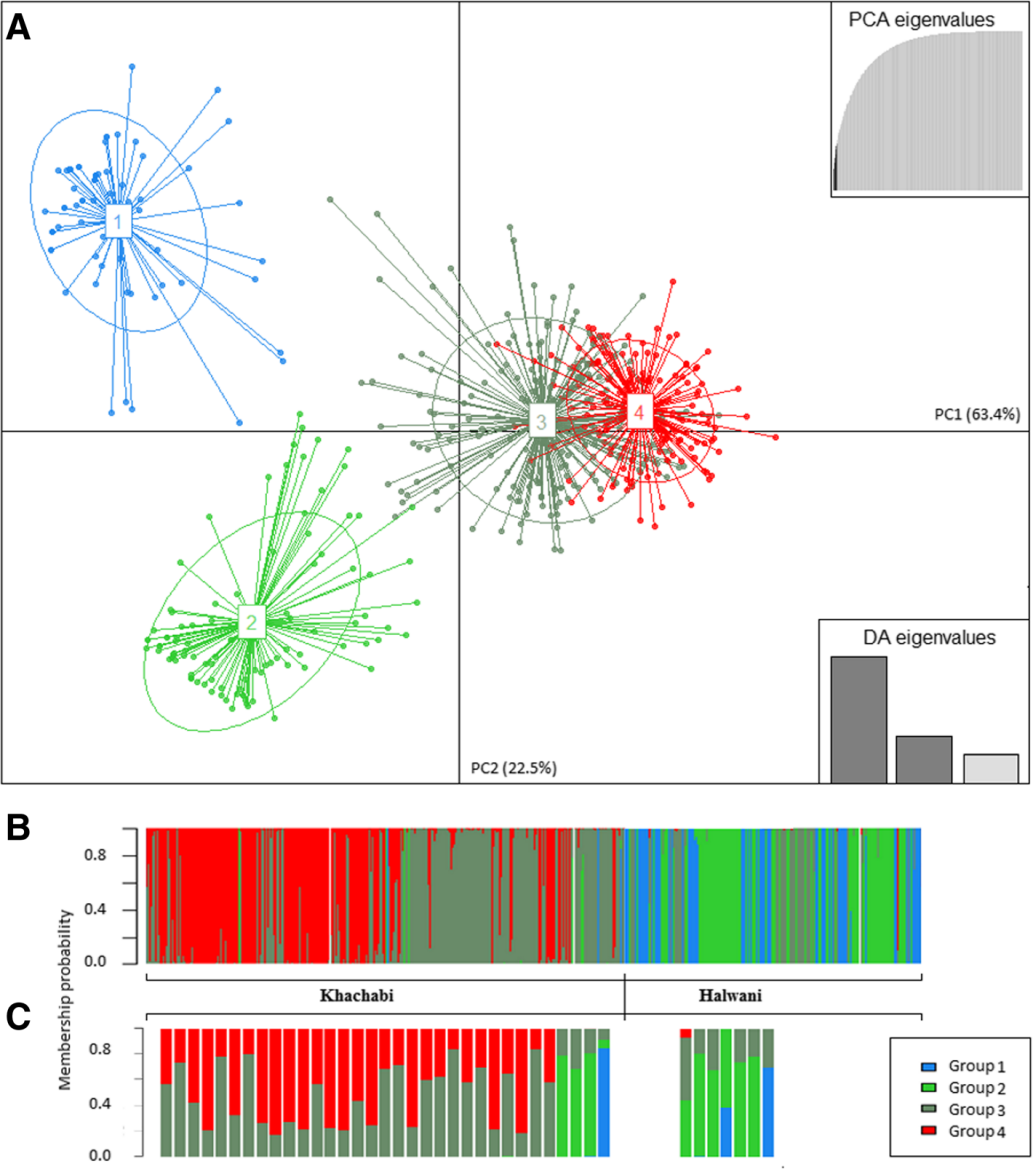
Genetic structure of almond genotypes estimated by DAPC. **a** Scatterplot of 509 MLGs on the first two axes representing 63.4% (PC1) and 22.5% (PC2) of the variation. Individuals are represents as dots and the groups as inertia ellipses. **b** STRUCTURE-like plot of 509 MLGs; **c**) Admixed individuals having no more than 85% of probability of membership in a single group. Each line corresponds to a single individual; colors correspond to the group memberships

The likelihood-based analysis of population structure using DAPC split the 509 MLGs from the 25 populations into four distinct groups (Fig. 5a and b). The ‘Khachabi’ MLGs were assigned to P3 (150 MLGs) and to P4 (153 MLGs), except for four MLGs assigned to the P1 and seven to P2 groups (Table 3). At a threshold value of 85% of assignation, a small number of individuals showed a pattern of admixture: 10 among P3, 19 among P4, 4 among P1 and 5 among P2 (Fig. 5c). The ‘Halwani’ MLGs were assigned into two more differentiated DAPC groups (Table 3): P1 (63 MLGs) comprised mainly individuals from the HM_Ma, HN_Bc and HB_Fe populations; P2 included 94 MLGs from all populations except HB_Ze, 37 MLGs were assigned to P3 and one MLG to P4. With the threshold value of 85% of assignation, only two MLGs of the P1 and three of the P2 were admixed. Admixed individuals were identified within and among cultivars (Fig. 5c). Within cultivars, 29 ‘Khachabi’ individuals were admixed between P3 and P4 groups while one ‘Halwani’ individual was admixed between P1 and P2 groups. Among cultivars, one ‘Khachabi’ individual was admixed among P1, P2 and P3, three MLGs were admixed between P2 and P3, one ‘Halwani’ MLG from HS_Ta was admixed between P1 and P3, another MLG was admixed among P2, P3 and P4, and four other ‘Halwani’ MLGs were admixed between P2 and P3.

**Table 3:** Assignment of 509 sampled individuals to STRUCRTURE’s clusters and DAPC’s groups

Structure	C1		C2		Admix^a^ between C1 and C2				G
DAPC	P3	P4	P1	P2	P1	P2	P3	P4	
HB_Bi			3	1			3		7
HB_Fe	1		12	2		1(1)^a^			16
HB_NA	7		4	4		4(1)^a^			19
HB_Ze			7						7
HM_Am				24		3			27
HM_Ma			13(1)^a^	8					21
HM_Je	14			4		6	5		29
HN_Bc	2		13	6			1		22
HN_Bt			1(1)^a^	11	1	8(1)^a^	2		23
HS_DQ		1	2	11					14
HS_Ta			5	1	2		2(1)^a^		10
KB_Ba	8(3)^a^	18(4)^a^							26
KB_Bi		20							20
KB_EM	2	27(2)^a^		1					30
KB_Ir	11	4(1)^a^							15
KB_KL	1(1)^a^	27(1)^a^							28
KB_Lu	6(1)^a^	24(3)^a^							30
KB_Sh	3(1)^a^	13(3)^a^	1	1					18
KN_As	13(1)^a^			1		1	1		16
KN_EQ	13(1)^a^	1					2		16
KS_Ba	28(1)^a^	1					1		30
KS_Bl	18(1)^a^	2(2)^a^					8	1	29
KS_Ch	7	14(1)^a^							21
KS_MJ	6					3(2)^a^	8		17
KS_Ta	5		1(1)^a^		2(1)^a^		9	1(1)^a^	18
Total	145	152	62	75	5	26	42	2	509

^a^ < 85% of alleles were assigned to a cluster and/or group

x (y), x: number of assigned individuals; y: number of admixture within assigned individuals, resulting from DAPC analysis

G, Number of distinct genotypes

The results from both Bayesian and non-model based analyses were compared at a threshold of 85% of assignment, for the global dataset (both cultivars; Table 3). Among ‘Khachabi’ individuals, 78%were assigned to cluster C1 according to STRUCTURE and to the P3 and P4 DAPC groups. As for ‘Halwani’, 66.7% of individuals assigned to cluster C2 according to STRUCTURE were assigned to the P1 and P2 DAPC groups.

Considering cultivars separately, the ‘Khachabi’ C1.1 and C1.2 STRUCTURE clusters correspond to P3 and P4 DAPC groups, respectively. As to ‘Halwani’, individuals assigned to C2.1 by STRUCTURE were assigned to P3 by DAPC; individuals assigned to the C2.2 and C2.3 STRUCTURE clusters were mainly assigned to the P1 and P2 DAPC groups, respectively.

## Discussion

Understanding how human practices affect the evolutionary dynamics of domesticated plant species is a key factor to improve the management of crop genetic diversity. It also helps understand how specific biological parameters, like reproductive systems and gene flow, affect genetic diversity. The cultivated almond is intermediate between seed propagated and vegetatively propagated perennial species; it provides a good model to answer our inquiries on domestication and diversification of vegetatively reproduced plants cultivated for their fruits and particularly self-incompatible fruit trees. Indeed, almond has been domesticated through seed selection and reproduction [9]; grafting was developed only over the last centuries; and its self-incompatibility system interferes with its sexual reproduction, as well as with fruit production per se. We will discuss our results concerning: 1) the high genetic diversity level of ‘Khachabi’ and its lower level in ‘Halawani’; 2) the deficit of heterozygosity in ‘Khachabi’ and its excess in ‘Halwani’; 3) the high genetic differentiation among ‘Halwani’ and ‘Khachabi’; 4) the high genetic differentiation between ‘Halwani’ groups; 5) the low genetic differentiation between both ‘Khachabi’ groups.

### Genetic diversity

As expected from the propagation mode of each cultivar and the outcrossing mating system of the species, the sexually propagated cultivar Khachabi exhibited much more genetic and genotypic diversity than the vegetatively propagated cultivar Halwani.

The level of genetic diversity observed within ‘Khachabi’ (He = 0.86) was as high as what was observed in national germplasm collections of different Mediterranean countries [19, 20, 22]. This genetic diversity could even be compared to the genetic diversity of spontaneous *Prunus* species, such as *P. orientalis* sampled from Lebanon, Syria and Turkey (He = 0.85) [26]. The comparison holds also at the population level. Indeed, in all seed-propagated ‘Khachabi’ populations, genetic diversity ranged between 0.74 and 0.84. These values compare with those observed in 20 wild Chinese populations of *P. sibirica* L., another self-incompatible species, where He ranged from 0.66 to 0.80 [27]. In our sample, the genotypic diversity level was only reduced for the ‘Khachabi’ KS_Ta population (*R* = 0.59), where the farmer had selected, and graft-propagated, three trees selected for their fruits. Even so, this unusual situation did not affect the level of allelic richness, as the selected individuals had retained a level of heterozygosity as high as for the seed-propagated ‘Khachabi’ populations.

‘Halwani’ exhibited a lower genetic diversity than ‘Khachabi’; which is due to the introduction of a limited number of individuals followed by clonal propagation. However, genetic diversity of ‘Halwani’ populations is still relatively high (He from 0.42 to 0.59), compared to cultivated self-pollinated *Prunus* species, as in the examples of three genetic groups of commercial peach cultivars collected from North America and Europe (He from 0.36 to 0.43) [38] and two genetic groups of sweet cherry cultivars collected from 16 countries and maintained in France (He values of 0.27 and 0.30, respectively) [62]. We relate this relatively high diversity to self-incompatibility in cultivar Halwani. Indeed, if orchards were constituted by a single self-incompatible genotype, flower fertilization would be hampered and commercial fruit production reduced. The only solutions are either to maintain pollen flow from external sources, or to maintain a minimal diversity of self-incompatibility alleles within ‘Halwani’ orchard. The first solution is observed when ‘Khachabi’ or spontaneous almond trees are present in the close vicinity, so they can participate in pollination. The second one implies a conscious choice of genetically diverse grafting materials, to avoid mate limitations within ‘Halwani’ orchards. Indeed, all ‘Halwani’ populations presented a composite structure with different genotypes. These management practices are found in cultivars of other *Prunus* species. For example, similar traditional management has long been known in cultivated apricot, where much of the variability has been conserved using a mix between seed- and graft-propagation (e.g. Vesuvian, Roussillon, and Peloponnese apricots) [63].

‘Khachabi’ exhibited a high heterozygosity deficit at both cultivar and population levels. These deficits can be explained in part by the presence of null alleles in seven loci, whose frequencies were significantly different from zero. Null alleles lead to underestimate the observed heterozygosity within populations and overestimate the fixation index [64]. It is likely that, on the long term, some level of endogamy (continued crosses among trees within population) has also contributed to this deficit.

The heterozygote excesses observed in ‘Halwani’ populations were in accordance with expectations; as repeated clonal propagation has preserved the initial heterozygosity of founder individuals. Similar results were observed for the perennial outcrossing crop cassava in Brazil [65], for improved apricot in Spain [66] and for *Prunus avium* in three French sites [8]. Over generations, somatic mutations may also contribute to increase heterozygosity. Such mutations were evidenced in pairwise comparisons among collected individuals: some MLGs were differentiated from others at only one of the fifteen analyzed loci, and by slight inter-allelic variations (1 or 2 bp).

### Genetic differentiation

The exploration of genetic structure by using both Bayesian and non-model methods has provided complementary information, by identifying different levels of genetic structure within the overall dataset (Fig. 5, Tables 3). The first, global, STRUCTURE analysis differentiated among cultivars while DAPC analysis detected structure among and within cultivars. The latter sub-structuring was also obtained when carrying out the STRUCTURE analysis for each cultivar separately. It is worth noting that the value of the statistic ΔK for K = 2 was large enough to approve the robustness of these clusters, and exclude the effect of some departure from STRUCTURE model hypotheses that may lead to a difficult estimation of the true number of clusters [67]. The assignment of individuals to DAPC groups was consistent with their assignment to the Structure clusters; we observed only a slightly lower consistency in the identification of admixed individuals between both methods.

Clustering results were in good accordance with cultivar denominations; the two major gene pools (STRUCTURE clusters C1 and C2) correspond to the studied cultivars, and the assignation of individual trees to one of them mostly reflects their denomination by farmers. Nevertheless, history of orchard establishment and rejuvenation has produced some discordance between farmers’ denominations and the genetic identity of some trees: 30 ‘Halwani’-named trees, from three different orchards, are assigned to the ‘Khachabi’ (C1) gene pool. This could be due either to the presence of ‘Khachabi’ trees in the ‘Halwani’ planting material coming from the nursery or to a failure of ‘Halwani’ grafting onto ‘Khachabi’ old trees when rejuvenating the orchard.

Dominant clonal propagation of cultivar Halwani has led to limited gene flow with ‘Khachabi’. Only four ‘Khachabi’ and six ‘Halwani’ individuals were not assigned to any groups by DAPC, and the detection of admixture suggests that these 10 genotypes were produced by hybridization. This low level of admixture was the result of a combination of both sexual and clonal propagation practices: sowing spontaneous hybrids between both cultivars, followed by clonal propagation of selected hybrid trees; this ensured bi-directional gene flow between cultivars. This effect of the propagation mode on the genetic differentiation was also documented for cultivated apricot: even though apricot in Tunisia has experienced a single introduction event from the Irano-Caucasian area, it has been subject to local diversification as a result of different propagation practices that have led to the existence of two different gene pools, one propagated by seeds and the other one propagated by grafting [21].

Our results demonstrate also that ‘Khachabi’ is structured into two relatively close gene pools (Fst = 0.033) separated geographically by the chain of Lebanese mountains; they correspond mainly to populations of the central plateau of the Bekaa region and its back mountains, for the first gene pool, and populations of the maritime range of coast and adjacent mountains, for the second gene pool. Admixed individuals were observed between these two gene pools. Farmers mainly established their ‘Khachabi’ orchards with seeds from nearby orchards or mountains (presumably ancient areas of cultivation). However, the presence of individuals from both gene pools, as well as admixed individuals, in the same orchards, attests for events of long distance seed migration among different geographic regions (Fig. 4a). Recurrent hybridizations between ‘Khachabi’ gene pools as shown by the sigmoidal shape of proportion of assignation among both ‘Khachabi’ clusters have weakened their differentiation. Likewise, seed migrations and hybridizations among individuals from nearby populations have contributed to reduce the effects of isolation by distance among collected populations. The first ‘Khachabi’ gene pool (C1.1) had no admixed individuals with either of the ‘Halwani’ gene pools. The clonal propagation of ‘Halwani’ and its recent introduction in the Bekaa region have prevented hybridizations. The second ‘Khachabi’ gene pool shares the same geographic range as the ‘Halwani’ cultivar, and longtime coexistence and sexual reproduction have resulted in admixture between cultivars.

As to cultivar Halwani, our results indicate that two different gene pools (Fst = 0.152) were introduced. This double origin was supported by the high values of Fst and Dc observed between some ‘Halwani’ populations. The first gene pool (C2.3) is mainly present in old orchards (Table 1). These orchards included more admixed individuals, confirming that propagation after theses early introductions has involved sexual recombination. The second gene pool (C2.2) is mainly present in the newest orchards (established in the 1980’s and 1990’s according to farmer interviews) where almond grafting was more intensively used. Therefore, it is not surprising that we observe only one admixture event between both gene pools, as time has been too short for consequent hybridization, particularly as concomitant evolutions of farmer’s practices have more clearly favored vegetative propagation over sexual recombination in the C2.2 gene pool of this cultivar. However, some pairs of geographically distant orchards, assigned to the same gene pool, exhibited low genetic distances, suggesting that a network of exchanges between farmers was effective on a large scale and/or that farmers have brought clonal plant material from a common source.

### Evolution of cultivated almond

The genetic diversity of the almond cultivars collected from this center of diversification revealed patterns of evolution, genetic structure, and geographical and social factors that have affected the crop in its evolutionary dynamics.

#### Evolutionary dynamics of ‘Khachabi’

In ‘Khachabi’, genetic structure patterns were similar to those observed for wild tree species. However, gene flow is not random among ‘Khachabi’ populations. Almond pollination is mainly carried out by bees. Although, in case of winter food scarcity, they can carry pollen between trees at distances up to 5 km [68], such long distance pollen exchanges are exceptional since this cultivar and other cultivated almond trees are present everywhere. Instead, local dispersal of seeds (by farmers or animals) and pollen explain the limited differentiation among neighboring populations and the correlation between genetic and geographic distances that we have observed (see Additional file 6: Figure S3); this is particularly true among the populations of the western Bekaa and the nearby Chebaa in the South (KS_Ch, Figs. 2 and 4). A limited isolation-by-distance is also consistent with a low level of endogamy and the consequent deficit in heterozygosity.

#### Dynamics of the integration of ‘Halwani’ grafted almond, and the adoption of grafting practices

‘Halwani’ was introduced from two different gene pools and propagated mainly in the coastal region. Both gene pools were introduced into the coastal regions by single or multiple introductions. The earliest one was concomitant with the incorporation of grafting practices for almond. Based on case studies from our data, we can envisage a consistent scenario explaining the evolutionary dynamics of ‘Halwani’, and particularly its interactions with ‘Khachabi’. When ‘Halwani’ was introduced in a given orchard, individuals of ‘Khachabi’ were preserved, leading to hybridization between both cultivars and the advent of admixed MLGs. Indeed, non-admixed ‘Halwani’ material sampled in the oldest ‘Halwani’ orchard (HM_Je) was only represented by three individual trees. As the subsequent phase of diffusion of ‘Halwani’ was based on locally collected materials, admixed materials could be part of the process. Thus, in the HB_Bi orchard, even though the farmer was sure to have ‘Halwani’ trees (having bought grafted trees at the local nursery), our genetic analysis identified trees with admixed MLGs resulting from hybridization between both cultivars. The presence of clones with admixed MLGs in the same geographic region further supports a scenario of propagation from nurseries. Another likely source of error in the diffusion of ‘Halwani’ was grafting failure, followed by ‘Kachabi’ rootstock suckering. This may be suspected for the HB_Na orchard; the farmer had bought his plants from a local nursery as ‘Halwani’, but the genetic analysis assigned many of them to ‘Khachabi’.

Later on, the grafting technique was better adopted. The success of grafting increased, explaining the marked predominance of clones in the C 2.2 gene pool that was more recently introduced to the Bekaa region (HB_Ze and HB_Fe orchards). The reduction of on-farm sexual propagation and the corollary appropriation of almond grafting practices led to a reduction of the genetic diversity of ‘Halwani’. Furthermore, farmers do not propagate all clones at the same rate, and in the long term, some clones will be lost.

In addition to the effect of farmers’ practices, networks of exchange have played an important role in structuring genetic diversity. Indeed, gene flow appeared to result from a network of exchanges of grafting material when establishing new orchards. The ‘Halwani’ grafts may have been taken from nurseries and/or orchards. This history of introduction and the exchange network of both ‘Halwani’ gene pools deserves to be explored more in depth in order to better understand the impact of human practices on the dynamics of this cultivar and its interactions with more ancient local germplasm.

#### Evolution of domesticated almond and propagation practices

Our comparative study of the two main Lebanese almond cultivars and their interactions provided us with the main elements of a model of evolution of self-incompatible perennial fruit crops under domestication and traditional cultivation. Furthermore, the comparison allowed us to describe the genetic effects of the concomitant introduction of a new cultivar and the corresponding propagation techniques. Grafting techniques are recent in cultivated almond evolution, and the first phases of its domestication process only involved allogamous sexual propagation; the late adoption of vegetative propagation limits the interest of the parallels often established with the domestication of crops derived from wild relatives that propagate vegetatively, such as cactus pear, banana and pineapple.

As long as allogamous sexual propagation is exclusive, genotypes cannot be fixed, and the genetic structure of cultivated populations remain similar to that of the fruit crop wild relatives, showing high genetic diversity within populations, limited differentiation among populations, and isolation by distance and topography. Thus, the genetic parameters reflecting the genetic organization of cultivar Khachabi populations in Lebanon compare excellently with those of close wild relatives.

The introduction of clonal propagation through grafting techniques brings the advantages of clonal propagation, particularly the fixation of elite genotypes, as well as other genetic effects, and first the reduction of genotypic diversity and a marked differentiation among populations. These effects are not as rapid and dramatic as could be expected, because of the long generation time that maintains old genetic patterns, and because farmers adapt their practices progressively, introducing vegetative reproduction in sexually propagated cultivars, as well as sexual recombination in the new clonal cultivars, thus generating new genetic diversity. As the evolution of farmers’ practices gives more and more prevalence to vegetative reproduction, newly introduced populations of the clonal cultivar evolve differently, with a stronger loss of genetic diversity and less interactions with ancient cultivars. The observed evolution of the cultivar Halwani follows clearly such a pattern.

The almond self-incompatibility system plays a particular role in these developments, imposing outcrossing in traditional, sexually propagated, cultivars, favoring recombination among ancient cultivars and new introductions, but also imposing a threshold of minimal genetic diversity to avoid mate limitation and the attendant yield reductions. Beyond this threshold, the system would evolve towards the exclusive use of vegetative propagation, as in western horticulture, implying the adoption of the complete technical package of modern horticulture (establishment of polyclonal orchards or use of specialized pollinator cultivars, unless a truly self-compatible cultivar is developed), while resulting in a dramatic erosion of genetic diversity.

## Conclusion

The population approach has allowed us to identify the effect of practices of farmers and nurserymen on the dynamics of genetic diversity within and among cultivars. Our results have shown different patterns of genetic diversity and population genetic structure for almond cultivars cultivated in Lebanon. Our study does not only contrast the effects of seed- and graft- propagation on genetic and genotypic diversity; it also shows that these propagation modes are not exclusive: farmers introduce clonal propagation in the seed-propagated cultivar while they maintain a diversity of genotypes within populations that are mostly graft-propagated. In such a mixed system, genetic diversity is not negatively affected and orchard productivity is not limited by the availability of compatible pollinators.

Our study also detected long-distance exchanges of planting materials. Elucidating the social networks driving them will lead to a better understanding of the spatial and temporal dynamics of the genetic structure of the Lebanese almond and of the diversification processes at work. It will provide more in-depth information to develop a strategy for in situ conservation of cultivars and to reduce gene flow from introduced material to ancient orchards, as well as to natural populations of various *Prunus* species.

## Additional files

Additional file 1: **Figure S1.** The geographic locations of the collected populations. A total of 14 ‘Khachabi’ and 11 ‘Halwani’ populations were sampled, covering the four major agro-climatic zones. (TIF 925 kb)

Additional file 2: **Table S1.** Statistics associated with microsatellite loci. Na, total number of alleles; PIC, polymorphic information content; Ho, observed heterozygosity; He, unbiased expected heterozygosity; Fis, inbreeding index calculated based on a matrix of N individuals or G genotypes; f (Null), estimation of significant frequency of null alleles. (a) Cipriani et al. 1999; (b) Testolin et al. 2000; (c) Sosinski et al. 2000; (d) Dirlewanger et al. 2002; (f) Aranzana et al. 2002. * HW significance with Bonferroni correction (*P* < 0.01). Fis _(N)_ values were calculated with all trees found including repeated clone genotype. Fis _(G)_ values were calculated with only one individual per multi locus genotype (MLG). (XLS 28 kb)

Additional file 3: **Table S2.** Genotypic linkage disequilibrium for pairwise comparisons between loci conducted over different data sets without redundant MLGs. – data not available; The coefficient of correlation is indicated for *P* > 0.05. (XLS 45 kb)

Additional file 4: **Figure S2.** Mean number of alleles per locus as a function of sample size. N, number of MLGs. Diamonds for global dataset, squares for ‘Khachabi’, triangles for ‘Halwani’. (TIF 36 kb)

Additional file 5: **Table S3.** Confidence intervals for Fis values at 95%. Fis calculated for sample size over 15 individuals (N) or genotypes (G). (XLS 25 kb)

Additional file 6: **Figure S3.** Plot of genetic distance (Dc) and geographic distance for A) 14 ‘Khachabi’ populations; B) 11 ‘Halwani’ populations. Significance at α = 0.05. (TIF 23 kb)

## Abbreviations

DAPC: Discriminant analysis of principal components
Dc: Genetic distance
ENA: Excluding null alleles
Fis: Wright’s fixation index
Fst: Genetic differentiation index
G: The number of distinct sampled genotypes
Ho: Observed heterozygosity
HWE: Hardy-weinberg equilibrium
IBD: Isolation by distance
INA: Including null alleles
K: Number of clusters
LD: Linkage disequilibrium
MCMC: Markov chain Monte Carlo
MLGs: Multi locus genptypes
N: The number of sampled individuals
PCR: Polymerase chain reaction
PI: Probability of identity
PIC: Polymorphic information content
R: Genotypic richness
SSR: Simple sequence repeat
UHe: Unbiased expected heterozygosity
