# Supplementary figures and images for: Evolution of almond genetic diversity and farmer practices in Lebanon: impacts of the diffusion of a graft-propagated cultivar in a traditional system based on seed-propagation
Source: BMC Plant Biol. 2018 Aug 6;18:155. doi: 10.1186/s12870-018-1372-8 (PMC6080396; doi:10.1186/s12870-018-1372-8)

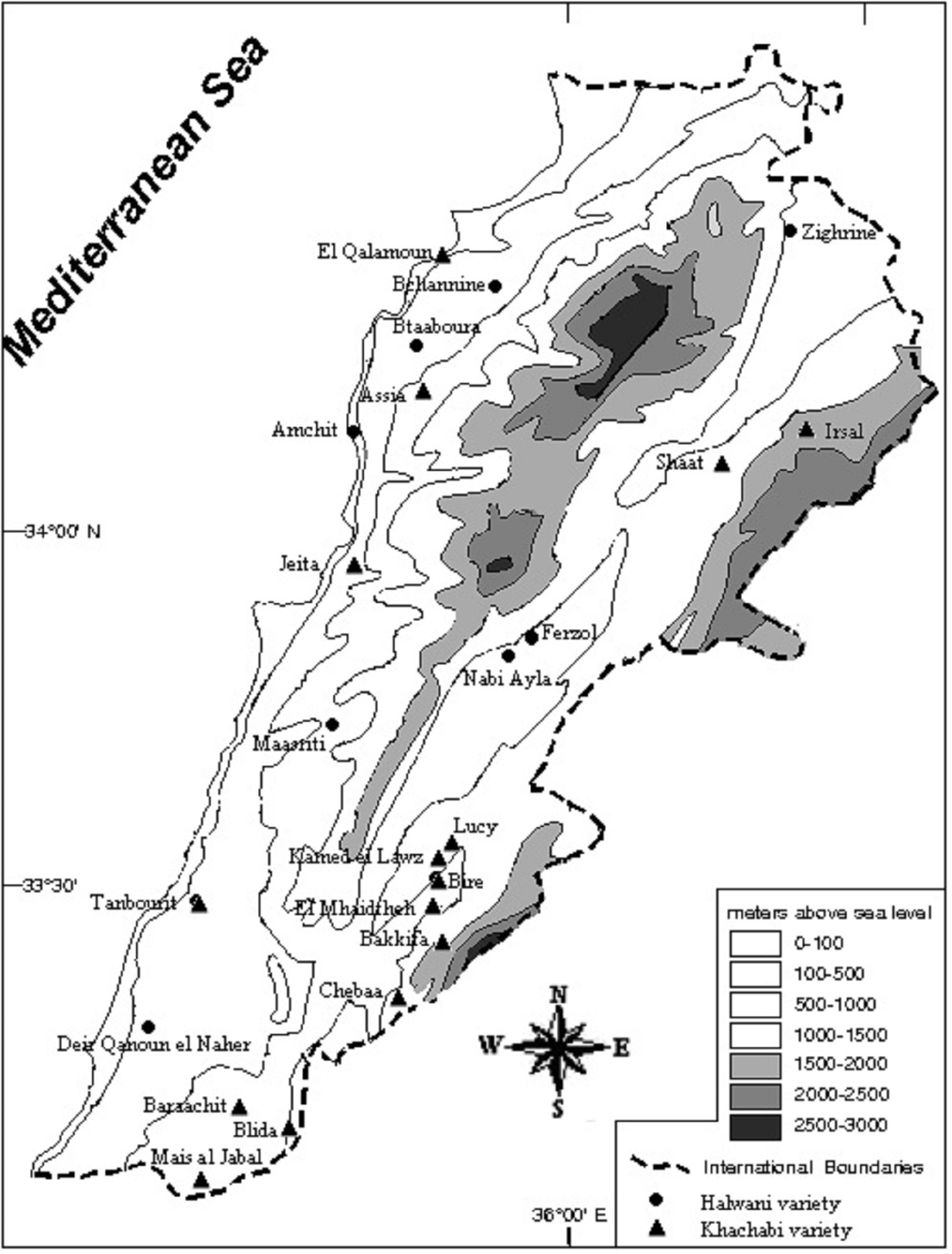

Supplement: Supplementary file 1 — Figure S1. The geographic locations of the collected populations. A total of 14 ‘Khachabi’ and 11 ‘Halwani’ populations were sampled, covering the four major agro-climatic zones. (TIF 925 kb) [file 12870_2018_1372_MOESM1_ESM.tif]

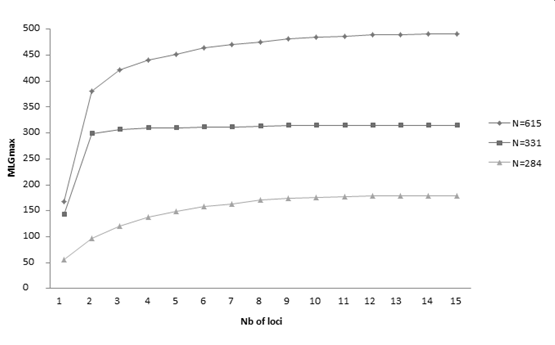

Supplement: Supplementary file 4 — Figure S2. Mean number of alleles per locus as a function of sample size. N, number of MLGs. Diamonds for global dataset, squares for ‘Khachabi’, triangles for ‘Halwani’. (TIF 36 kb) [file 12870_2018_1372_MOESM4_ESM.tif]

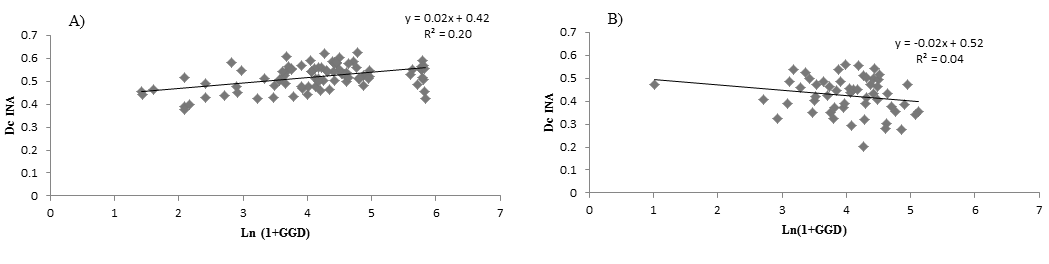

Supplement: Supplementary file 6 — Figure S3. Plot of genetic distance (Dc) and geographic distance for A) 14 ‘Khachabi’ populations; B) 11 ‘Halwani’ populations. Significance at α = 0.05. (TIF 23 kb) [file 12870_2018_1372_MOESM6_ESM.tif]
